# Cost-effectiveness of different interferon beta products for relapsing-remitting and secondary progressive multiple sclerosis: Decision analysis based on long-term clinical data and switchable treatments

**DOI:** 10.1186/2008-2231-21-50

**Published:** 2013-06-22

**Authors:** Shekoufeh Nikfar, Abbas Kebriaeezadeh, Rassoul Dinarvand, Mohammad Abdollahi, Mohammad-Ali Sahraian, David Henry, Ali Akbari Sari

**Affiliations:** 1 Department of Pharmacoeconomics and Pharmaceutical Administration, Faculty of Pharmacy, Tehran University of Medical Sciences, Tehran, Iran; 2 Food & Drug Organization, Ministry of Health & Medical Education, Tehran, Iran; 3 Faculty of Pharmacy, and Pharmaceutical Sciences Research Center, Tehran University of Medical Sciences, Tehran, Iran; 4 Department of Neurology, Sina Hospital, Tehran University of Medical Sciences, Tehran, Iran; 5 Institute for Clinical Evaluative Sciences, Toronto, Canada; 6 Department of Health Management and Economics, School of Public Health, Tehran University of Medical Sciences, Tehran, Iran

**Keywords:** Cost-Effectiveness, Decision analysis, Economic evaluation, Interferon beta, Markov model, Modeling, Switching

## Abstract

**Background:**

Multiple sclerosis (MS) is a highly debilitating immune mediated disorder and the second most common cause of neurological disability in young and middle-aged adults. Iran is amongst high MS prevalence countries (50/100,000). Economic burden of MS is a topic of important deliberation in economic evaluations study. Therefore determining of cost-effectiveness interferon beta (INF β) and their copied biopharmaceuticals (CBPs) and biosimilars products is significant issue for assessment of affordability in Lower-middle-income countries (LMICs).

**Methods:**

A literature-based Markov model was developed to assess the cost-effectiveness of three INF βs products compared with placebo for managing a hypothetical cohort of patients diagnosed with relapsing remitting MS (RRMS) in Iran from a societal perspective. Health states were based on the Kurtzke Expanded Disability Status Scale (EDSS). Disease progression transition probabilities for symptom management and INF β therapies were obtained from natural history studies and multicenter randomized controlled trials and their long term follow up for RRMS and secondary progressive MS (SPMS). A cross sectional study has been developed to evaluate cost and utility. Transitions among health states occurred in 2-years cycles for fifteen cycles and switching to other therapies was allowed. Calculations of costs and utilities were established by attachment of decision trees to the overall model. The incremental cost effectiveness ratio (ICER) of cost/quality adjusted life year (QALY) for all available INF β products (brands, biosimilars and CBPs) were considered. Both costs and utilities were discounted. Sensitivity analyses were done to assess robustness of model.

**Results:**

ICER for Avonex, Rebif and Betaferon was 18712, 11832, 15768 US Dollars ($) respectively when utility attained from literature review has been considered. ICER for available CBPs and biosimilars in Iran was $847, $6964 and $11913.

**Conclusions:**

The Markov pharmacoeconomics model determined that according to suggested threshold for developing countries by world health organization, all brand INF β products are cost effective in Iran except Avonex. The best strategy among INF β therapies is CBP intramuscular INF β-1a (Cinnovex). Results showed that a policy of encouraging accessibility to CBPs and biosimilars could make even high technology products cost-effective in LMICs.

## 

Multiple sclerosis (MS) is a highly debilitating immune mediated disorder of the central nervous system [1]. Since MS is a complicated illness to diagnose accurately, the worldwide variation in prevalence and incidence is not precisely known. The best estimate is that around 2.5 million people in the world suffer from MS [2]. The range of prevalence estimates of MS in different countries and regions differs from 5 to 189 per 100,000 [3,4]. In a report published in 2000, Iran as a Middle Eastern country was listed among low to medium zone incidence of MS based on the theory of geographical epidemiology [4]. The prevalence rate of MS is estimated to be 50 per 100,000, translating to around 35,000 cases (Table 1) [5-17]. Therefore, Iran can be considered to be amongst high MS prevalence countries [4]. The onset of MS usually occurs during early adulthood (age 15–45 years) [18] making MS the second most common cause of neurological disability in young and middle-aged adults [19]. In Iran, the mean age for incidence and prevalence of MS are 27 and 32, respectively with a 2.8 times higher incidence in women than that of men. The most recent census of the Iranian population (2011) shows a youth bulge in the age range of 20 to 29 years old [20]. This means that health providers have to be ready to face the MS burden and its economic consequences.

**Table 1:** Epidemiologic details of published studies of multiple sclerosis (MS) in Iran

**Study**	**Number of patients**	**Location**	**Prevalence/ 100000**	**Incidence rate/ 100000**	**Female/Male ratio**	**Age (mean ± SD)**	**Age of onset (mean ± SD)**	**Education (High school graduated & University education %)**	**Occupational State (Employed %)**
**Yousefi Pour et al., 2002**[5]	142	Fars	5.3	-	1.2:1	32.7±6.5	-	-	-
**Kalani et al., 2003**[6]	200	Tehran, (Loghman hospital)	-	-	2.5:1	-	27±7.4	95%	-
**Saadatnia et al., 2007**[7]	1718	Isfahan	43.8	3.64	-	-	25.36±8.6	-	-
**Hashemilar et al., 2011**[8]	1000	East Azarbayjan	27.7	-	2.7:1	33.4	-	-	-
**Abedini et al., 2008**[9]	582	Mazandaran	20.1	-	2.6:1	34.4±9.4	26.9±8.3	-	24.6%
**Milo and Kahana, 2010**[10]	-	Iran	44	-	-	-	-	-	-
**Sahraian et al., 2010**[11]	8146	Tehran	51.9	-	2.6:1	-	27.74±8.32	-	-
**Etemadifar et al., 2006**[12]	1391	Isfahan	-	-	3.6:1	32.5	-	50.9%	30.8%
**Elhami et al., 2011**[13]	7896	Tehran	50.57	2.93	3.11:1	-	-	-	-
**Nabavi et al., 2006**[14]	203	Tehran (Shahid Mostafa Khomeini hospital)	-	-	1.5:1	35.6	-	-	30.2%
**Ghandehari et al., 2010**[15]	800	Khorasan (Razavi, Northern, Southern)	9.86	-	1.6:1	-	-	-	-
**Ghabaae et al., 2007**[16]	70	Tehran (Emam Khomeini hospital)	-	-	2:1	32.58±10.24	27.55±10.42	39.2%	-
**Ale-Yasin et al., 2002**[17]	318	Iran	-	-	1.52	35.4±9.6	26.6±8.1	73.2%	

Several immunomodulatory treatments including interferon beta (INF β), glatiramer acetate and natalizumab and one immunosuppressive treatment (mitoxantrone) have been approved for MS patients with a relapsing course [21]. Three preparations of recombinant IFN β have been approved for use in MS, subcutaneous IFN β-1a (SC IFN β-1a), intramuscular IFN β-1a (IM IFN β-1a), and subcutaneous IFN β-1b (SC IFN β-1b). IFN β is indicated for the treatment of relapsing-remitting form of MS to reduce the frequency of clinical exacerbations and delay the development of physical disability [22]. According to a previous meta-analysis, INF β’s effectiveness in MS varies with the different kinds of INF β and the types of MS. Generally, IFN β can control remission in MS but its effectiveness in secondary progressive multiple sclerosis (SPMS) and relapsing remitting multiple sclerosis (RRMS) is questionable [22,23]. On other hand, the use of the immunomodulatory therapies in clinical practice has been a topic of substantial debate concerning clinical and cost-effectiveness. Although interferon has not been recommended formally by The National Institute for Clinical Excellence (NICE) in the UK many clinical practice guidelines have recommended the immunomodulatory therapies for the treatment of MS [18].

Based on the experience of using INF β in treatment of patients with MS for about 20 years in the Europe and United States, it is considered expensive but modestly effective in terms of reduction of morbidity and improving the quality of life [24-26]. However, it is a challenge for most countries to afford new expensive therapies for MS within limited public resources [27]. Although studies have shown considerable differences between high-income and low- or middle-income countries in capacity to afford the flow of costly new therapies, INFs β have been in use in Iran since 1995, starting with SC INF β-1b and there has been an exponential increase in use. Since 2009, the ‘copied biopharmaceuticals’ (CBPs) and biosimilars versions of INF β with lower and more affordable prices have been registered and have become accessible in Iran [28,29].

Iran as an Asian country located in the Eastern Mediterranean region and a middle-income country has some specific demographic, economic and health indicators that are presented in Table 2[30]. Iran is one of the oldest countries in the region that adopted a National Drug Policy (NDP) many years ago [31]. On the basis of the NDP, a specific National Drug List was established to regulate strategies and to ensure registration of only high-quality effective drugs for Iranian citizens at the lowest possible price, mostly by use of subsidization [32,33]. To implement this goal, the Iranian Food and Drug Organization has the mission to control the price of medicines and balance their usage on the basis of the established NDP [34] by considering cost efficacy and equity of access [35].

**Table 2:** Demographic, economic and health indicators of Iran

**Total Population**	73,974,000
**Gross national income per capita (PPP) ($)**	11,490
**GDP/capita ($)**	5810
**Life expectancy at birth male/female(years)**	70/75
**Total expenditure on health per capita ($, 2010)**	836
**Total expenditure on health as % of GDP (2010)**	6.8
**Distribution of years of life lost by causes (2008)**	
**Communicable/Noncommunicable/Injuries**	% 28/49/23

$: International Dollars.

Cost-effectiveness and cost-utility analyses (CEA/CUAs) have been used increasingly in the last two decades by funders to judge the balance between the added expenses of new drugs and their incremental benefits (e.g., improved patient outcomes). The majority of published CEA/CUA reports on INF β therapies for MS come from studies carried out in high income countries [18,19,36-41]. Economic evaluations based on decision analytic modeling are an alternative to trial-based economic evaluations. The use of decision analytic models in economic evaluations is the only framework that has the potential to meet all the requirements for economic evaluation for decision making and to ensure their applicability in countries of varying national wealth [42]. Markov models are particularly useful when the clinical setting involves a risk that is ongoing over time.

The cost-effectiveness of INF β treatments has been estimated by Markov models in previous studies, but these have ignored differences of effectiveness and tolerability of different INF β products. Previous studies done by us have highlighted the varying efficacy of different kinds of INF β in RRMS or SPMS [22,23]. When there is a probability of switching to other drugs in a decision analysis model, overhead costing may affect results and decision-making processes [35]. Thus in this study we consider results due to switching to other drugs.

The main goal of this study was to perform CEA/CUA evaluations using a decision analysis model of INF β therapies for MS from the perspective of a low-middle-income countries (LMICs). We examined the cost-effectiveness of 4 treatment strategies in patients diagnosed with RRMS (symptom management alone and in combination with IM IFN β-1a, SC IFN β-1a, or SC IFN β-1b).

## Methods

### Description of the model

**Figure 1:**
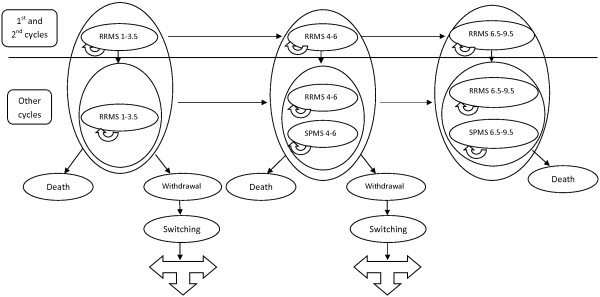
Schematic Representation of the Markov Model and decision tree for evaluation of INF β cost-effectiveness in MS.

A new Markov model was set up to assess the cost-effectiveness of 4 treatment strategies to manage a hypothetical cohort of patients diagnosed with RRMS in Iran. A decision analysis approach was used to model the effects of switching treatments (Figure 1). The incremental cost-effectiveness analysis was the main analytical plan for the study, combining cumulative measures of costs over time with a cumulative measure of effectiveness. This approach evaluates the incremental costs per clinical benefit gained (expressed as cost per QALY gained). The analysis was performed for a hypothetical cohort of 30-year-old patients. The inclusion criteria used in the randomized controlled trials (RCTs) of INF β was taken into account and the effectiveness was noted as QALYs. The model embraced patterns of resource utilization in both outpatient and inpatient care. Also, both direct non-medical and indirect costs were included.

The treatment strategies were management of diseases symptoms alone and in combination with one of the following medicines: IM IFN β-1a, SC IFN β-1a, or SC IFN β-1b. The clinical course of RRMS (e.g., disease progression and transition to SPMS) was modeled in terms of the Kurtzke expanded disability status scale (EDSS) and withdrawal from treatment [43]. Specifically, 7 health states were modeled (Figure 1):

1. RRMS-EDSS 1–3.5: no or few limitations in mobility

2. RRMS-EDSS 4–6: moderate limitations in mobility

3. RRMS-EDSS 6.5-9.5: walking aid or wheelchair required and restricted to bed

4. SPMS-EDSS 4–6: moderate limitations in mobility

5. SPMS-EDSS 6.5-9.5: walking aid or wheelchair required and restricted to bed

6. Death (natural causes or EDSS 10)

7. Withdrawal due to side effects and perceived lack of efficacy or other causes like pregnancy and financial problems

Transitions among the health states occurred in 2-years cycles. A 2-years cycle time was used, because this interval closely approximates the follow-up period of the IFN β RCTs for preventive treatment in RRMS. Most of information for SPMS was reported in 3 year intervals; thus they were calculated for 2 years to input to the model. The baseline time horizon of the model was assumed to be a lifetime in order to capture the full benefits of immunomodulatory therapy, which was assumed to be around 30 years after onset of the disease (15 cycles). Costs and outcomes were estimated from the societal perspective and were discounted at 7.2% per annum for cost [44] and 3% for quality of life [45]. The costs and QALYs were discounted from the end of first year. All the costs were reported in 2012 U.S. dollars and exchange rates of IRR to U.S. dollars of 12260. Data were obtained from literature covering RCTs of IFN β, official prices, and tariff lists (see below for details). To validate the methodology (model structure and assumptions), four expert opinion leaders were consulted.

The model calculated the following outcomes: numbers of patients remaining in lower EDSS; numbers of patients remaining in RRMS; numbers of patients remaining relapse free; QALYs; total costs and costs by component (i.e., IFN β therapy cost, MS-related medical costs [e.g., drugs for symptom management], and lost worker productivity costs; direct non-medical costs and incremental cost-effectiveness ratios (ICER) comparing symptom management alone with symptom management combined with each of the 3 IFN β therapies. Given that there is no accurate threshold calculated for Iran, the cost/QALY ICERs were compared with multiples of the gross domestic product (GDP) per capita according to recommendations of the World Health Organization (WHO) [46]. According to the WHO the treatments of MS with different IFN β would be considered “highly cost effective” if the cost/QALY is less than GDP per capita, “cost-effective" if the cost/QALY was between one to three times of GDP per capita and “not cost-effective” if it was more than three times of GDP per capita. The GDP per capita of Iran was considered as 5810 USD [30]. Model parameters were varied in sensitivity analyses.

A number of underlying assumptions were adopted for the base-case model:

1. In the model, all patients start in the health state stage of EDSS 1–3.5.

2. The point at which patients transitioned from RRMS to SPMS was assumed to be in the third cycle (approximately 5 years after diagnosis of illness) and the model assumed that this took place between EDSS 4–6 and EDSS 6.5-9.5 [47]. Corresponding to this assumption, the model assumed that relapses occurred in patients didn’t change the state of EDSS [48].

3. The model assumed that as per product labeling and off-labeled prescriptions, only RRMS and SPMS patients in EDSS of 1–6 were eligible for and received IFN β therapies.

4. Switching among the IFN β therapies was permitted once in the model. Patients who withdrew IFN β therapy during the first three cycles were assigned the transition probabilities for relapse and disease progression used in the symptom management arm or other two IFN β arms. Patients who discontinued therapy were not permitted to reinitiate therapy. Switching between IFN β was acceptable within brands or biosimilar and CBPs only.

5. In case of withdrawal from IFN β therapy in cycles of 4 to 15, patients were allocated to the transition probabilities for relapse and disease progression used in the symptom management arm.

6. It was presumed that there was no variation in mortality between patients with or without preventive treatment [22] and there was no sex-related mortality risk factor in RRMS and SPMS [49].

7. Other direct medical and non-medical costs and indirect costs were calculated according to utilization in different status and stages of disease and the cycle of Markov model.

8. Medicinal treatments and laboratory tests for management of MS morbidity in different groups were deemed according to results of our previous meta-analysis [22]. Cost of antidepressants, anti-spasmodics, anti-fatigues, pain killers, and NSAIDs were considered in our model.

9. Weighted mean costs were considered for other medicines consumed by MS patients in both generic or brand forms. Frequency of use was based on survey data [28].

10. Calculations were performed for cost of hospitalization, physicians’ visits, laboratory tests, imaging, psychotherapy and physiotherapy according to percentages of patients using governmental and private facilities or outpatient admission to daycare clinics.

### Clinical and economic outcomes

The effectiveness measurement was based on the concept of utility, which measures the QALYs [50].

The cost assessment was based on the assignment of costs to the health states and interventions.

### Data sources

Three different types of data can be distinguished in modeling studies:

1. Probabilities of clinical events: progress of disease, chance of an acute exacerbation, withdrawal, and death.

2. Utilities of different Markov health states.

3. Costing information derived from estimates of the units of resource utilization and their prices/tariffs (product of unit and price).

4. Probabilities of switching to other INF β or symptomatic treatments.

**Figure 2:**
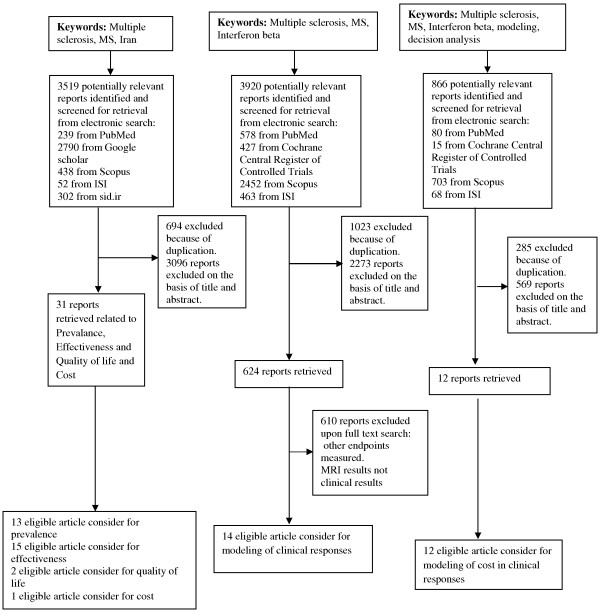
Search diagram for Multiple sclerosis (MS), Interferon beta (INF β) and Iran.

To design the strategy of retrieving data from PubMed, Scopus, Web of Science, and the Cochrane library, Iranmedex, SID, and MagIran were searched for studies reporting efficacy and/or tolerability of IFN β in multiple sclerosis, and epidemiology, natural history and costs of MS. Data were collected from 1966 to 2012 (up to December). The search terms were: “multiple sclerosis” or “MS” and “IFN beta” or “Interferon beta”; “multiple sclerosis” or “MS” and “Iran”; “multiple sclerosis” or “MS” and “IFN beta” or “Interferon beta” and “modeling” or “decision analysis”, “cost”. The language was restricted to English and Persian. The reference lists from retrieved articles were also reviewed to avoid missing any relevant studies (Figure 2).

The probabilities of clinical events, including disease progression, probability of an acute exacerbation, and withdrawal were based on IFN β clinical trial data and their long term data. A cross sectional study has been developed to evaluate cost and utility. An approved questionnaire was adopted and validated. Two-hundred MS patients were recruited consecutively in a random manner from three referral hospitals of two different cities and three private offices of MS specialists and members of Iranian society of MS through society’s office in Tehran and Arak and patients’ email [51]. Evaluation of recruitment showed no bias (p>0.05) according to feature of MS retrieved through systematic review (Table 3). ANOVA statistical test for normal numerical variables was used to detect differences in the groups retrieved from literature search and recruited group corrected by Bonferroni post hoc test.

**Table 3:** Baseline characteristics of patients recruited to evaluate quality adjusted life years QALYs and Cost for multiple sclerosis (MS) in Iran (n = 200)

	**Total recruited MS patients**	**RRMS**			**SPMS**		**Unknown or PPMS**
		**EDSS**			**EDSS**		
		**1-3.5**	**4-6**	**>6**	**4-6**	**>6**	
**Numbers of Patients**	200	104	29	12	24	4	PPMS: 19 Unknown: 8
**Age (mean±SD)**	33.8±9.1	29±7.7	36±7.2	36.6±9.2	42.4±6.7	46±3.7	PPMS: 41.8±6.3 Unknown: 34.5±8.2
**Sex (Female/Male)**	148/52	80/24	24/5	10/2	16/8	2/2	PPMS: 10/9 Unknown: 6/2
**Urban/Rural**	174/26	93/11	25/4	12/0	20/4	4/0	PPMS: 13/6 Unknown 4/4
**Education (High school graduated ** **& University education %)**	83%	83%	79%	83%	83%	100%	PPMS: 53% Unknown: 75%
**Occupational State (Employed %)**	31%	37%	48%	0%	29%	0%	PPMS: 0% Unknown: 13%

*MS* multiple sclerosis, *RRMS* relapsing remitting multiple sclerosis, *SPMS* secondary progressive multiple sclerosis, *PPMS* primary progressive multiple sclerosis, *EDSS* expanded disability status scale.

Age and sex-specific mortality rates assumed that MS alters life expectancy and increases the rate of death threefold across the different age or sex groups (Table 4) [49]. Age and sex-specific population mortality rates were derived from national statistics data [52].

**Table 4:** Age and sex adjusted mortality rate for multiple sclerosis (MS) patients

**Age (range)**	30-35	36-40	41-45	46-50	51-55	56-60	61-65	66-70
**Mortality rate (%)**	7.69	7.61	8.29	10.17	12.71	14.19	16.2	18.9

Probabilities were derived from published literature (Table 5): Relapse and disease progression transition probabilities within the symptom management arm were obtained from published natural history studies [47,53,54].

**Table 5:** Probability of related outcomes to multiple sclerosis (MS) and interferon beta (INF β) therapy

	**Natural History**		**Interferon beta-1a**		**Interferon beta-1a 44 Mcg/0.5 ml**		**Interferon beta-1b**	
			**(Avonex)**		**(Rebif)**		**(Betaferon)**	
**Probability of EDSS progression within RRMS**								
RRMS 1-3 to RRMS 3.5-6	0.4		0.219		0.193		0.2	
RRMS 3.5-6 to RRMS 6≤	0.27		0.11		0.115		0.15	
**Probability of progression from RRMS to SPMS (the same EDSS)**									
RRMS 3.5-6 to SPMS 3.5-6	0.43		0.43		0.197		0.433		
RRMS 6≤ to SPMS 6≤	0.31		0.31		0.31		0.31		
**Probability of EDSS progression within SPMS**									
SPMS 3.5-6 to SPMS 6≤	0.5		0.3		0.5		0.4		
**Relapse rate**									
RRMS	Cycle 1	2.54	Cycle 1	1.34	Cycle 1	1.73	Cycle 1	1.68	
	Other cycles	2.04	Other cycles	1.31	Other cycles	1.18	Other cycles	1.01	
SPMS	0.7		0.47		0.5		0.35		
**Withdrawal**									
RRMS 1-3	-		Cycles1 &2	0.04	Cycles1 &2	0.078	Cycles1 &2	0.086	
			Other cycles	0.04	Other cycles	0.05	Other cycles	0.09	
RRMS 3.5-6	-		Cycle 2	0.26	Cycle 2	0.42	Cycle 2	0.247	
			Other cycles	0.04	Other cycles	0.05	Other cycles	0.09	
SPMS 3.5-6	-		0.19		0.357		0.25		

*RRMS* relapsing remitting multiple sclerosis, *SPMS* secondary progressive multiple sclerosis, *EDSS* expanded disability status scale, *Mcg* microgram, *ml* milliliter.

Treatment Effects of the IFN β treatment effects were obtained from RCTs (IM IFN β-1a, SC IFN β-1a, SC IFN β-1b) and long-term follow-up studies [55-65].

The QALYs for the different health states were derived from a cross-sectional study as shown in Table 6. Utility estimates were based on direct elicitation methods of visual analogue scale (VAS) and generic preference-based measures of the EuroQol (EQ-5D) and the Health Utilities Index 3 (HUI3) by in-house translated and validated questionnaires. We also derived utility loss per EDSS and relapse from literatures [19,66,67].

**Table 6:** Utility scores related to multiple sclerosis (MS) type and expanded disability status scale (EDSS) states in cost-effectiveness model

**State**	**Utility score**			
	**Literature review**	**Visual Analog Scale (VAS)**	**European Quality of Life-5 Dimensions (EQ-5D)**	**Health Utility Index 3 (HUI 3)**
**RRMS EDSS 1-3.5**	0.68	0.79	0.76	0.68
**RRMS EDSS 4-6**	0.52	0.62	0.57	0.41
**SPMS EDSS 4-6**	0.52	0.58	0.52	0.18
**RRMS EDSS 6<**	0.17	0.38	0.18	0.08
**SPMS EDSS 6<**	0.17	0.18	0.02	0.06
**Relapse (Utility loss)**	0.5	0.42	0.42	0.42
**Death**	0	0	0	0

All utility scores have been obtained from cross section study based on questionnaire.

Other necessary information were obtained indirectly by calculations that were done on the basis of long-term follow up data, hazard rate, variances of Kaplan–Meier curves [68,69] and/or by solving multiple-step equations.

Probabilities of switching to other INF βs or no treatment in case of withdrawal due to side effects or lack of perceived efficacy were derived from the cross sectional study.

### Costs

Data on costs were derived from cross sectional study (Table 7).

**Table 7:** Cost of multiple sclerosis disease (MS) and interferon beta (INF β) therapy

**Cost per patient per year ($)**		**RRMS 1-3.5**	**RRMS 4-6**	**RRMS 6<**	**SPMS 4-6**	**SPMS 6<**
		**Total costs for other medications is related to” Interferons-beta” administration and duration, EDSS and type of disease**				
**Total medical direct cost (Medicines)**						
Interferon beta-1a (Avonex)*		9861	9861	0	9861	0
Interferon beta-1a (Rebif)*		7106	7106	0	7106	0
Interferon beta-1b (Betaferon)*		9176	9176	0	9176	0
Interferon beta-1a IM (CBPs)*		3762	3762	0	3762	0
Interferon beta-1a SC (CBPs)*		5873	5873	0	5873	0
Interferon beta-1b (CBPs & BS)*		7969	7969	0	7969	0
Antidepressant medications	The first 3 cycles	2.3	2.7	3.2	2.7	3.2
	Other Cycles	1.5	1.8	2.2	1.8	2.2
Anti-spasm medications		11.65	11.65	11.65	11.65	11.65
Anti-fatigue medications		1.36	1.36	1.36	1.36	1.36
Pain killer medications		4.7	6.6	9.8	23.5	29.9
NSAIDs (control SE)	for Interferon beta-1a IM	0.15	0.15	0	0.15	0
	for Interferon beta-1a SC	0.42	0.42	0	0.42	0
	for Interferon beta-1b	0.53	0.53	0	0.53	0
**Other medical direct cost (total)**		**167**	**316**	**1543**	**316**	**1543**
Laboratory tests		13	13	0	13	0
Imaging		103	103	0	103	0
Physicians visits		45	32	14	32	14
Physiotherapy		0	15	326	15	326
Psychotherapy		6	64	0	64	0
Nursing		0	41	816	41	816
Cane, walker, wheelchair, Medical bed, Medical wave mattress		0	48	387	48	387
**Non-medical direct cost (total)**		**266**	**1841**	**3037**	**1841**	**3037**
Transport		266	373	40	373	40
House reconstructions		0	0	1529	0	1529
Car rebuilding		0	1468	1468	1468	1468
**Indirect cost (total)**		**90**	**130**	**616**	**379**	**740**
Absence from work		71	120	0	65	0
Unemployment or early retirement		7	0	616	308	740
Mortality		12	10	0	6	0
**Cost per patient per relapse (total)**		**33**	**33**	**33**	**54**	**54**
Medical treatment		27	27	27	45	45
Hospitalization		6	6	6	9	9

*$* US Dollars 2012, *IM* intramuscular, *SC* subcutaneous, *NSAIDs* non steroid anti- inflammatory drugs, *Cost of injection is included for interferons beta; *CBPs* Copied biopharmaceuticals including IM IFNβ-1a (Cinnovex, Actovex), SC IFNβ-1a (Recigen, Actorif ) and SC IFNβ-1b (Ziferon, Actoferon), *BS* biosimilar including SC IFNβ-1b (Extavia).

We used a backward-looking approach in which resource utilization and clinical data were gathered at a single point in time and covered the one-year period prior to the dates of inclusion. The cost of care of MS was calculated for patients in current clinical practice in Iran in 2012 for each state of disease. The costs due to relapses derived from the cost-of-care study in same cross sectional study and evaluating of patients files archived in the hospitals. All prices were extracted from official list of tariffs [70,71]. The friction cost method was used to evaluate workday’s loss by on the go patients [72]. By use of this method, the significance of productivity loss was assumed to be 80% of the ordinary rate of employee’s productivity during the “friction period.” Thereafter, it was assumed that sick employees could be replaced. Time lost by inactive patients was considered as leisure time lost and was valued at 40% of the average wage, productivity and friction period in Iran [20,73,74].

### Sensitivity analysis

Elementary effects sensitivity analyses were done according to basic clinical and economic assumptions and their changes in the clinical-outcome model. This was used to test the stability of the suppositions of the analysis over a range of assumptions, probability estimates, and value judgments. The first sensitivity analysis was performed to assess the impact of CBPs and biosimilars on the analysis. We performed the second analysis to assess the sensitivity of the model to different ways of QALYs assessments obtained from questionnaires and literatures’ searches-based QALYs. The third sensitivity analysis was performed to evaluate the sensitivity of analysis to discount cost and QALYs.

### Consent

The study was approved in the Institute Review Board with code number 91-10-24:1–1. Additionally, the study was approved by Iranian Society of MS. Written informed consent was obtained from the patient for the publication of this report and any accompanying images.

## Results

### ICER of symptom management combined with IFN β comparing to symptom management alone

ICER of adding IM IFNβ-1a (Avonex) to symptom management based on evaluation of utility by literatures search, VAS, EQ-5D, and HUI3 as measurement tools of QALYs was $18712, $19954, $17398 and $20045. Not cost-effective intervention except while evaluated by EQ-5D.

ICER of adding SC IFNβ-1a (Rebif) to symptom management based on evaluation of utility by literatures search, VAS, EQ-5D, and HUI3 was $11832, $11850, $10433 and $11437 (cost-effective intervention).

ICER of adding SC IFNβ-1b (Betaferon) to symptom management based on evaluation of utility by literatures search, VAS, EQ-5D, and HUI3 was $15768, $16864, $15030 and $16314 (cost-effective intervention).

ICER of adding CBPs IM IFNβ-1a (Cinnovex, Actovex with 98 and 2 percent of market share among CBPs IM IFNβ-1a ) to symptom management based on evaluation of utility by literatures search, VAS, EQ-5D, and HUI3 as a measurement tools of QALYs was $847, $904, $788 and $908 (highly cost-effective intervention).

ICER of adding CBPs SC IFNβ-1a (Recigen, Actorif with 87.5 and 12.5 percent of market share among CBPs SC IFNβ-1a) to symptom management based on evaluation of utility by literatures search, VAS, EQ-5D, and HUI3 was $6964, $6975, $6140 and $6731 (cost-effective intervention).

ICER of adding CBPs SC IFNβ-1b (Ziferon, Actoferon with both 12.5 percent and (biosimilar); Extavia with 75 percent of market share among CBPs and biosimilar SC IFNβ-1b) to symptom management based on evaluation of utility by literatures search, VAS, EQ-5D, and HUI3 was $11913, $12740, $11355, and $12325 (cost-effective intervention). All results have been provided in Table 8.

**Table 8:** ICER ($) of different Interferons-Beta in comparison to placebo therapy in MS patients according to different utility measurement methods

**Therapy**	**Literature-based**	**VAS**	**EQ-5D**	**HUI 3**
**Interferon beta-1a**	18712	19954	17398	20045
**(Intramuscular)**				
**(Avonex)**				
**Interferon beta-1a**	11832	11850	10433	11437
**(Subcutaneous)**				
**(Rebif)**				
**Interferon beta-1b**	15768	16864	15030	16314
**(Betaferon)**				
**Interferon beta-1a**	847	904	788	908
**(Intramuscular)**				
**(CBPs)**				
**Interferon beta-1a**	6964	6975	6140	6731
**(Subcutaneous)**				
**(CBPs)**				
**Interferon beta-1b**	11913	12740	11355	12325
**(CBPs and BS)**				

*$* US dollars, *ICER* incremental cost effectiveness ratio, *VAS* visual analogue scale, *EQ-5D* EuroQol, *HUI3*: Health Utilities Index 3, *CBPs* Copied biopharmaceuticals including IM IFNβ-1a (Cinnovex, Actovex), SC IFNβ-1a (Recigen, Actorif ) and SC IFNβ-1b (Ziferon, Actoferon), *BS* biosimilar including SC IFNβ-1b (Extavia).

### Discounted ICER of symptom management combined with IFNβ comparing to symptom management alone

Discounted ICER of adding IM IFNβ-1a (Avonex) to symptom management based on evaluation of utility by literatures search, VAS, EQ-5D, and HUI3 as a measurement tools of QALYs was $18873, $20370, $18050, and $20104 (not cost-effective intervention). 

Discounted ICER of adding SC IFNβ-1a (Rebif) to symptom management based on evaluation of utility by literatures search, VAS, EQ-5D, and HUI3 as a measurement tool of QALYs was $13482, $13885, $12347, and $13092 (cost-effective intervention).

Discounted ICER of adding SC IFNβ-1b (Betaferon) to symptom management based on evaluation of utility by literatures search, VAS, EQ-5D, and HUI3 was $15142, $16452, $14808, and $15663 (cost-effective intervention).

Discounted ICER of adding CBPs IM IFNβ-1a (Cinnovex, Actovex with 98 and 2 percent of market share among CBPs IM IFNβ-1a) to symptom management based on evaluation of utility by literatures search, VAS, EQ-5D, and HUI3 as a measurement tools of QALYs was $4026, $4345, $3850, and $4288(highly cost-effective intervention).

Discounted ICER of adding CBPs SC IFNβ-1a (Recigen, Actorif with 87.5 and 12.5 percent of market share among CBPs SC IFNβ-1a) to symptom management based on evaluation of utility by literatures search, VAS, EQ-5D, and HUI3 was $9553, $9838, $8749, and $9276 (cost-effective intervention).

Discounted ICER of adding CBPs SC IFNβ-1b (Ziferon, Actoferon with both 12.5 percent and (biosimilars) Extavia; with 75 percent of market share among CBPs and biosimilar SC IFNβ-1b) to symptom management based on evaluation of utility by literatures search, VAS, EQ-5D, and HUI3 was $11903, $12933, $11641 and $12312 (cost-effective intervention). All results have been provided in Table 9.

**Table 9:** Discounted ICER ($) of different Interferons-Beta in comparison to placebo therapy in MS patients according to different utility measurement methods

**Therapy**	**Literature-based**	**VAS**	**EQ-5D**	**HUI 3**
**Interferon beta-1a**	18873	20370	18050	20104
**(Avonex)**				
**Interferon beta-1a**	13482	13885	12347	13092
**(Rebif)**				
**Interferon beta-1b**	15142	16452	14808	15663
**(Betaferon)**				
**Interferon beta-1a**	4026	4345	3850	4288
**(Intramuscular)**				
**(CBPs)**				
**Interferon beta-1a**	9553	9838	8749	9276
**(Subcutaneous)**				
**(CBPs)**				
**Interferon beta-1b**	11903	12933	11641	12312
**(CBPs & BS)**				

*$* US dollars, *ICER* incremental cost effectiveness ratio, *VAS* visual analogue scale, *EQ-5D* EuroQol, *HUI 3* Health Utilities Index 3, *CBPs* Copied biopharmaceuticals including IM IFNβ-1a (Cinnovex, Actovex), SC IFNβ-1a (Recigen, Actorif ) and SC IFNβ-1b (Ziferon, Actoferon), *BS* biosimilar including SC IFNβ-1b (Extavia).

## Discussion

The results of these analysis showed that all kinds of available branded, CBPs or biosimilar IFN β in Iran are cost-effectiveness except Avonex. The analysis was based on Markov model and long-term data of effectiveness and tolerability and considering progress to SPMS. Switching is the noteworthy difference of this model that has been implemented for the first time for cost-effectiveness of IFN β in MS. WHO’s recommendation about threshold of developing countries considers ICER less than triplet of GDP as a cost-effective intervention [46]. GDP of 2012 for Iran is 5810 USD, thus all ICER less than 17430 USD per QALYs could be considered cost-effective. Analysis showed ICER of 847 to 16864 USD per QALYs for CBPs and biosimilar of IM IFNβ-1a, SC IFNβ-1a and SC IFNβ-1b, Rebif and Betaferon. For Avonex, intervention is cost-effective only when EQ-5D has been applied for assessment of utility. The differences of QALYs assessment tools in calculation of utility in MS have been demonstrated before [75]. It seems that this difference happened because EQ-5D questionnaire is not capable to measure one of the important disutility of MS, cognition. However sensitivity analysis showed that our model was not sensitive to evaluation methods of utility or literature-based ones. The model is not sensitive to discounting either. Our model is sensitive to replacing CBPs and biosimilars that it is a dominated approach. Sensitivity in case of bivariate analysis of discounting and CBPs IM IFNβ-1a and SC IFNβ-1a have occurred. This may happen because of significant impact of IFN β in cost of MS treatment. Therefore, reducing the price of IFN β by replacement with CBPs will decrease the influence of this factor and may highlight other reasons that affect utility. It must be emphasized that the unit price of CBPs IM IFNβ-1a and SC IFNβ-1a are significantly lower than Avonex and Rebif in Iran. Overall sensitivity analysis in this study showed robustness of this model.

This is the first time that cost-effectiveness of IFN β have been analyzed in context of one of developing countries by applying decision analytic modeling. Markov model has been applied to assess the progression of disease and its prevention with adding IFN β to management protocol. Spreading on the concept of switching to other IFN β in case of withdrawal due to side effects or lack in perceived efficacy to model is novel. The idea for modifying the Markov model in case of switchable interventions has been established by Nikfar 2012 [35]. In case of probability of switching to other drugs in decision analysis model, overhead costing and utility may affect results and decision-making processes, thus, for the first time we consider switching to other IFN β in case of withdrawal from one of them. Therefore, to conclude the utility and cost of each intervention, decision tree model has been used as an additional decision analytic modeling. Additional analysis showed that without considering switching to other IFN β, ICER will be beyond threshold and intervention will not be cost-effective.

In our Markov model, the probability of transition to SPMS is one of the transition states. Nuijten and Hutton have also considered SPMS in their Markov model [19]. Their analysis revealed that IFN β were much more cost-effective compared with the results of other cost-effectiveness study performed in patients with RRMS only. They believe that reduction in the cost-effectiveness ratio of IFN β may be in line for a longer follow-up period and continuation of treatment with IFN β in SPMS.

Our designed model is based on thirty-year that is kind of life-long follow up complying with life expectancy of MS patients that is almost 10 years less than healthy population [49]. Considering the rate of mortality in MS patients rather than general population [18,19,41], the reliability of study is increased. Seeing IFN β act in MS patients differently is in contrast with modeling of Bell et al., and Nuijten and Hutton [18,19] and robust the results of current cost-effectiveness study and may help decision makers accurately. It should be emphasized that assuming tolerability as an essential part of drug therapy that interferes with health-related outcomes [76] is another advantage of this study. Withdrawal due to side effects of medicines or lack of effectiveness in long-term treatments may have a great impact in cost-effectiveness analysis [77]. Chilcott et al. [41] didn’t consider withdrawal in their decision analysis model, although Nuijten and Hutton [19] and Bell and his colleagues [18] have used withdrawal data in their model. The withdrawal data was retrieved from short-term trials and only due to side effects [19] or lack of efficacy [18] and just for one cycle of model. To be closer to reality, withdrawal is considered in all long of intervention in recent model. Availability of long-term follow-up data (effectiveness and withdrawal) of patients initially enrolled in clinical trials [63-65] helped us to not repeating the mistake of previous studies in use of extrapolated data from short-term trials to assess effectiveness and discontinuation of therapy [19,41].

However, our model is much more improved but always the results of modeling need to be treated with some degree of caution from a health-economic perspective. Amongst is the data for probabilities of preventive effects for IFN β. All such kinds of data in this analysis have been retrieved from RCTs and their long-term follow up data. Therefore, the fact of controversy between real effectiveness and efficacy obtained from protocol-restricted RCTs will remain. Usually, conducting meta-analysis can solve this problem, but in case of rare diseases like MS due to lack of variety of available trials, this method is even unlikely. The indirect cost in developing countries may be affected by low productivity of such regions. Furthermore, Iran has adapted generic based pharmaceutical policy. These two reasons may consequence underestimation of the cost in this model and the low ICER than other cost-effectiveness studies [18,19,41,78] that reported approximately 50000 USD per QALYs, for prevention effect of IFN β. The utilities in our model were derived from a cross-sectional study based on EDSS and disease conditions. Adverse effects of therapeutics medicines have not been considered in assessment of utilities and this may also modify the results of ICERs. There are specific health related quality of life (HRQOL) questionnaires for MS like Hamburg Quality of Life Questionnaire in Multiple Sclerosis (HAQUAMS) or multiple sclerosis quality of life with 54 questions (MSQOL-54), moreover quality of life in MS can be also determined by generic HRQOL like EQ-5D or HUI-3. The problem of specific HRQOL is measurement of utility or preference-based measures by them that is impossible; on the other hand, utility or preference-based measures have the advantage of leading to a single number that balances gains in one domain against losses in another. The best known utility or preference-based measures according to reliability, validity and feasibility for MS are the HUI-3 and the EQ-5D index [79,80]. There are varieties in concepts and scores of utility measurement tools. It seems that HUI3 with potential of scoring cognitive problems of MS patients could act better than other tools like EQ-5D or VAS. Nevertheless, sensitivity analysis in our model didn’t show differences between utilities obtained from different forms and literature reviews. The characteristics of patients related to age, sex, educational and employment status were taken from 200 patients in cross sectional filled questionnaires. According to prevalence of different kind of MS and EDSS state, in some states like higher EDSS or SPMS there are too few recruited patients. This problem may underweight the results and considering equal and sufficient amount of patients for each state, in future studies is recommended to vigorous results. Our model with 50% progression to SPMS after 10 years and 90% after 25 years [47] and life expectancy of thirty after incidence of illness corresponds with natural history of MS [49].

Our results showed that prescribing of all kind of IFN β except Avonex in context of Iran is cost-effective. Due to the fact that there is no difference among IFN β as regards of effectiveness and safety, thus the choice of them depends on clinicians in many cases. However, it seems that price is an important factor for prescription. Therefore CBP of IM IFNβ-1a (Cinnovex) is prescribed more than 50% in Iran [28]. As a pharmaceutical regulatory rule, clinical data for brands and CBPs and biosimilar of IFN β in this study considered to be the same [28,81]. If by availability of post marketing surveillance, we reach the same conclusion about ranking for ICERs, the most efficient way is to choose the most cost-effective IFN β with respect of compliance.

The results of this study demonstrated that generic based NDP is one of the strength of each country’s policy to make medicines more accessible.

## Competing interests

The authors declare that they have no competing interests. Since MA is Editor-in-Chief of DARU, all review process of the submission was handled by one of Section Editors.

## Authors’ contributions

SN made design of the study, acquisition of data, analysis and interpretation of data, drafted the article and revising it critically for important intellectual content, AK and RD reviewed all data and supervised whole study, MA supervised whole study and revised the paper critically for important intellectual content, MAS contributed in acquisition of data and interpretation of data, DH contributed in design of the study, AAS reviewed all data and design of study and supervised whole study. All authors approved final version for submission.
